# Ara h 6 complements Ara h 2 as an important marker for IgE reactivity to peanut

**DOI:** 10.1186/2045-7022-3-S3-P168

**Published:** 2013-07-25

**Authors:** J Hindley, A Koid, RG Hamilton, R van Ree, SA Versteeg, SC Dreskin, MD Chapman, S Wunschmann

**Affiliations:** 1 INDOOR Biotechnologies Ltd, Cardiff, UK; 2 INDOOR Biotechnologies Inc, Charlottesville, VA, USA; 3 Johns Hopkins University School of Medicine, Baltimore, MD, USA; 4 Academic Medical Centre, Amsterdam, the Netherlands; 5 University of Colorado, Denver, CO, USA

## Background

Ara h 6 has a reported seroprevalence similar to Ara h 2, a major peanut allergen. Both allergens are of similar molecular size and share 50% identity in their amino acid sequences. Their similarity has made it difficult to obtain natural Ara h 6 free of Ara h 2 for immunoreactivity studies. The objective of this study was to obtain purified natural Ara h 6 that is essentially free of Ara h 2 and to compare its IgE reactivity and potency in histamine release assays to Ara h 2.

## Methods

Natural Ara h 6 was purified from peanut flour extract by affinity chromatography and size exclusion chromatography. Purified Arah 6 was analyzed by silver-stained SDS-PAGE and tandem mass spectrometry (LC/MS-MS). The immunological and biological reactivity of Ara h 6 and Ara h 2 were compared by ELISA and histamine release assays.

## Results

SDS-PAGE of the highly purified allergen revealed a single 14.5kD band and the identity of Ara h 6 was confirmed by tandem mass spectrometry (LC/MS-MS). The purified nAra h 6 contained <0.01% traces of Ara h 2 as assessed by ELISA. Natural Ara h 6 had a lower biological activity in basophil histamine release assays than natural Ara h 2. Chimeric ELISA showed that 70 and 75% of peanut allergic patients (n=57) had specific-IgE to natural Ara h 2 and natural Ara h 6 respectively.

## Conclusion

Ara h 6 is a major peanut allergen, with comparable immunoreactivity to Ara h 2. The highly purified Ara h 6, free of Ara h 2, will be useful for diagnostic IgE antibody assays, and for molecular and cellular studies to further investigate the immunological mechanisms of peanut allergy.

## Disclosure of interest

J Hindley: Employee of INDOOR Biotechnologies Ltd, A Koid: Employee of INDOOR Biotechnologies Inc, R Hamilton: None declared, R van Ree: None declared, S Versteeg: None declared, S Dreskin: None declared, M Chapman: Shareholder of INDOOR Biotechnologies Inc, S Wunschmann: Employee of INDOOR Biotechnologies Inc.

